# “Pink Pattern” Visualized in Magnifying Endoscopy With Narrow-Band Imaging Is a Novel Feature of Early Differentiated Gastric Cancer: A Bridge Between Endoscopic Images and Histopathological Changes

**DOI:** 10.3389/fmed.2021.763675

**Published:** 2021-11-15

**Authors:** Shengsen Chen, Jiangping Yu, Rongwei Ruan, Yandong Li, Yali Tao, Qiwen Shen, Zhao Cui, Cheng Shen, Huogen Wang, Jiayan Jin, Ming Chen, Chaohui Jin, Shi Wang

**Affiliations:** ^1^Department of Endoscopy, Zhejiang Cancer Hospital, Institute of Cancer and Basic Medicine, University of Chinese Academy of Sciences, Hangzhou, China; ^2^Hithink RoyalFlush Information Network Co., Ltd, Hangzhou, China

**Keywords:** pink pattern, vessel-plus-surface classification system, early gastric cancer, magnifying endoscopy, histopathological changes

## Abstract

**Background:** A pink color change occasionally found by us under magnifying endoscopy with narrow-band imaging (ME-NBI) may be a special feature of early gastric cancer (EGC), and was designated the “pink pattern”. The purposes of this study were to determine the relationship between the pink pattern and the cytopathological changes in gastric cancer cells and whether the pink pattern is useful for the diagnosis of EGC.

**Methods:** The color features of ME-NBI images and pathological images of cancerous gastric mucosal surfaces were extracted and quantified. The cosine similarity was calculated to evaluate the correlation between the pink pattern and the nucleus-to-cytoplasm ratio of cancerous epithelial cells. Two diagnostic tests were performed by 12 endoscopists using stored ME-NBI images of 185 gastric lesions to investigate the diagnostic efficacy of the pink pattern for EGC. The diagnostic values, such as the area under the curve (AUC), the accuracy, sensitivity, specificity, positive predictive value (PPV), and negative predictive value (NPV), of test 1 and test 2 were compared.

**Results:** The cosine similarity between the color values of ME-NBI images and pathological images of 20 lesions was at least 0.744. The median AUC, accuracy, sensitivity, specificity, PPV, and NPV of test 2 were significantly better than those of test 1 for all endoscopists and for the junior and experienced groups.

**Conclusions:** The pink pattern observed in ME-NBI images correlated strongly with the change in the nucleus-to-cytoplasm ratio of gastric epithelial cells, and could be considered a useful marker for the diagnosis of differentiated EGC.

## Introduction

Gastric cancer is one of the commonest cancers worldwide, and globally ranks fifth and fourth in morbidity and mortality, respectively. In 2020, the number of new cases exceeded 1 million and the estimated number of deaths was 769,000 (1). The early detection of gastric cancer is essential for a good prognosis. Magnifying endoscopy with narrow-band imaging (ME-NBI) is a new powerful optical imaging technique that has significantly improved endoscopic diagnoses when narrow-band filters are used (2). The microvascular architecture and microsurface structure of the gastrointestinal tract can be clearly visualized with ME-NBI (3). Therefore, ME-NBI has been widely used for the diagnosis of early gastric cancer (EGC) (4, 5).

Yao et al. analyzed images of gastric cancer taken with ME-NBI and proposed a gastric cancer diagnosis system called the “vessel-plus-surface (VS) classification system” (4, 6, 7). This system is particularly useful in distinguishing superficial differentiated gastric cancers of types 0–II, although it is still difficult to distinguish cancers with pale lesions (predominantly undifferentiated cancers and signet ring cell carcinomas) (8, 9). When the VS classification system is used for EGC diagnosis, the most important step is determining whether there is a demarcation line (DL) in the suspicious lesion. DL is defined as the boundary line between the cancerous area and the non-cancerous area. An obvious irregular microvascular pattern (IMVP) and/or irregular microsurface pattern (IMSP) can be seen within the DL (4, 8, 9). However, in the actual endoscopic examinations of differentiated EGC, the DL is sometimes indeterminate, or the DL is visible but the IMVP and/or IMSP are indeterminate (10). It is currently difficult to accurately discern a cancerous lesion with the VS classification system under ME-NBI. Therefore, it is imperative to find a new endoscopic feature of differentiated gastric cancer that allows endoscopists to more confidently diagnose EGC endoscopically when it is difficult to identify the DL or IMVP/IMSP according to the VS classification system.

**Figure 1:**
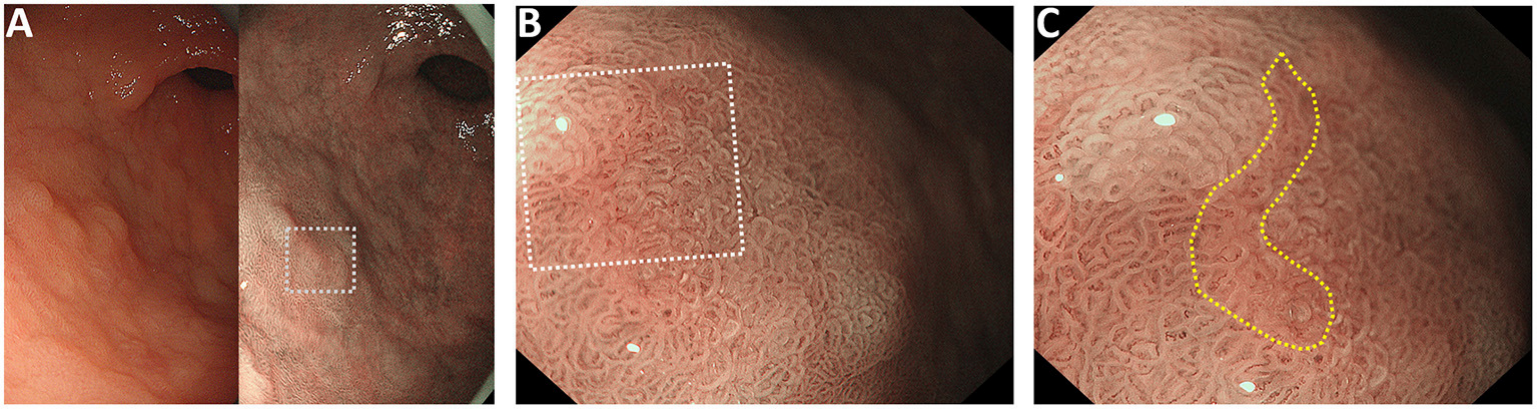
Representative case of the “pink pattern” when the demarcation line (DL) is indeterminate. **(A)** Suspected lesion of early cancer in the gastric antrum (dotted boxed area). Left panel shows the conventional white light imaging findings and the right panel shows the narrow-band imaging findings. **(B)** Magnified image of the dotted boxed area in **(A)**. DL is indeterminate. **(C)** Magnified image of the dotted boxed area in **(B)**. “Pink pattern” is observed in the lesion (within the yellow dotted line).

**Figure 2:**
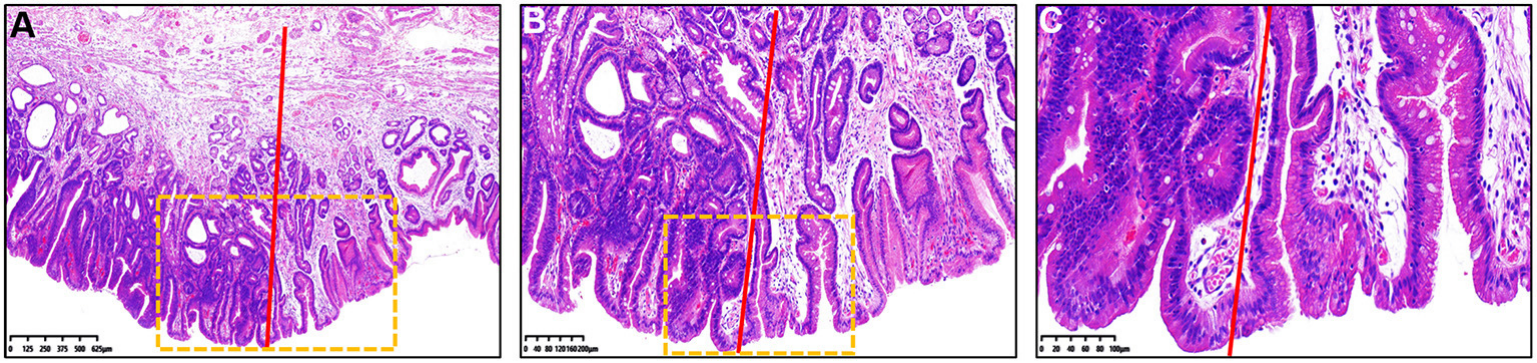
Histopathological findings of Figure 1 under different magnification. **(A–C)** Hematoxylin and eosin staining of the resected specimen showed that the histopathological diagnosis was gastric cancer. Color of the gastric epithelial cells to the left of the red line was much darker than that to the right of the red line. **(B)** Magnified image of the dotted boxed area in **(A)**. **(C)** Magnified image of the dotted boxed area in **(B)**. The nucleus-to-cytoplasm ratio of epithelial cells to the left of the red line was higher that of cells to the right of the red line.

When EGC is observed, the color of the gastric mucosal surface is usually masked by the surface microvessels, so any color changes in the superficial mucosal epithelium are easily missed. We have occasionally observed a pink color change on the superficial mucosal epithelium when suspicious gastric malignant lesions are visualized with ME-NBI, when the DLs of these lesions are either indeterminate or clear (Figure 1, Supplementary Figure 1). The lesions that showed a pink color change, regardless of whether the DL was indeterminate or visible, were confirmed as gastric cancer by histological examination (Figure 2, Supplementary Figure 2). We designated this pink color change in a gastric cancer lesion under ME-NBI the “pink pattern” (the additional images can be seen in Supplementary Figure 3). Interestingly, based on the further observation of histopathological slices, we found that the nucleus-to-cytoplasm ratio of the epithelial cells in these cancerous lesions was higher than that of non-cancerous lesions (Figure 2, Supplementary Figure 2). Therefore, we hypothesized that this pink pattern on ME-NBI images of gastric cancer lesions correlated strongly with the change in the nucleus-to-cytoplasm ratio of the gastric epithelial cells in these lesions on histopathological images.

In this study, we tested our hypothesis using a computer image-processing technique, and explored the pink pattern observed under ME-NBI to determine whether it is useful in the diagnosis of EGC. Twelve endoscopists examined stored ME-NBI images in two diagnostic tests. We propose a new efficient strategy for EGC diagnosis using ME-NBI.

## Methods

### Endoscopic System and Procedures

**Figure 3:**
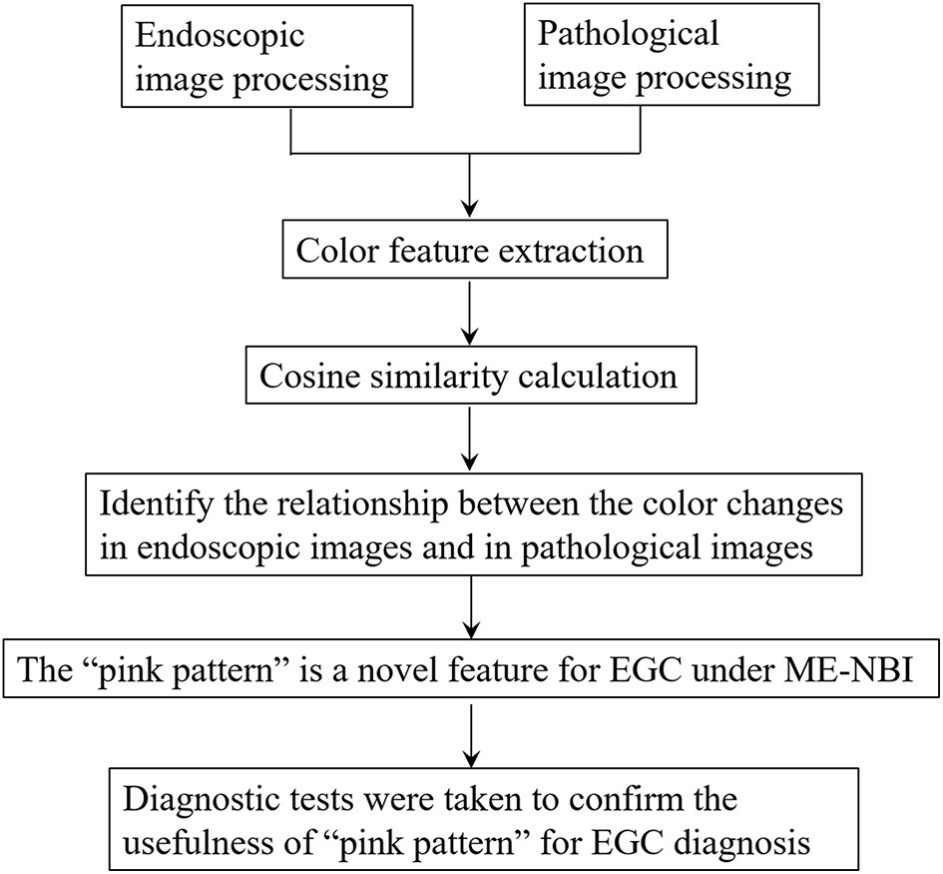
Flow chart of this study.

The flowchart for the present study is shown in Figure 3. All images were taken with an electronic endoscopy system with NBI (high resolution optical magnifying endoscopes, GIF-H290Z and GIF-H260Z; Olympus). First, endoscopic examinations were performed by endoscopists using white light imaging. After a suspicious lesion was detected, a magnified observation was made by fixing the NBI mode. A soft black hood was mounted on the tip of the scope, allowing the endoscopist to consistently fix the distance between the tip of the scope and the target lesion to ~2 mm. The endoscopic images were then obtained with maximal magnification. High quality NBI images of the lesions were selected for the subsequent diagnostic tests. A biopsy had not been taken from any lesion before the ME-NBI examination.

### Histopathological Examination

Endoscopic submucosal dissection specimens or biopsy specimens were fixed with 20% formalin for 24 h. The fixed tissues were then embedded in paraffin and sliced at 2 mm intervals, maintaining the thickness of the slices at 5 μm. The slices were stained with hematoxylin and eosin (HE) and examined histopathologically under a light microscope. Histopathological diagnoses were made with reference to the revised Vienna classification (C1 to C5) (11).

### Endoscopic Image Processing

#### Glandular Tube Mask Generation

Given an endoscopic image *I*, the Fast Global Smoother (12) was firstly used for the generation of the lightmap *L*. Then a Gamma correction was performed on *L* to adjust the light balance and brightness according the following equation:


$$\begin{array}{l} R=\frac{I}{L^{\lambda }} \end{array}$$

Where *L* is the lightmap, λ (λ* was setted as* 0.75) is the gamma value, and *R* is the corrected lightmap. To make the texture color difference between the crypt epithelium and the background more obvious, the grayscaling processing is performed on both image *I* and *R* to obtain ImgGray and ImgGSF, respectively. Then the texture enhancement image (ImgEnhance) was obtained with the following equation:


$$\begin{array}{l} \text{ImgEnhance}=\text{ImgGray}-\text{ImgGSF}. \end{array}$$

Finally, we used the Otsu thresholding algorithm (13) to extract the Glandular Tube Mask (GTM) from ImgEnhance.

Based on the generated GTM, a skeleton extraction algorithm (14) and a GrabCut algorithm (15) were combined to refine the GTM. Firstly, the GTM was dilated as a possible foreground GTM0 and eroded as a definite foreground GTM1. Secondly, the pixel values of GTM0 and GTM1 were set to 3 and 1, respectively, and a new mask GTM2 was created by the overlay of GTM0 and GTM1. A new GTM3 was finally generated by applying GrabCut algorithm (15) on GTM2.

In consideration of color distortion in the endoscopic image, the Gray World algorithm (16) was used to correct the color of the endoscopic image and then the endoscopic image is filtered to remove the image noise with Gaussian Blur function (17).

#### Feature Point Sampling and Saturation Curve Drawing

**Figure 4:**
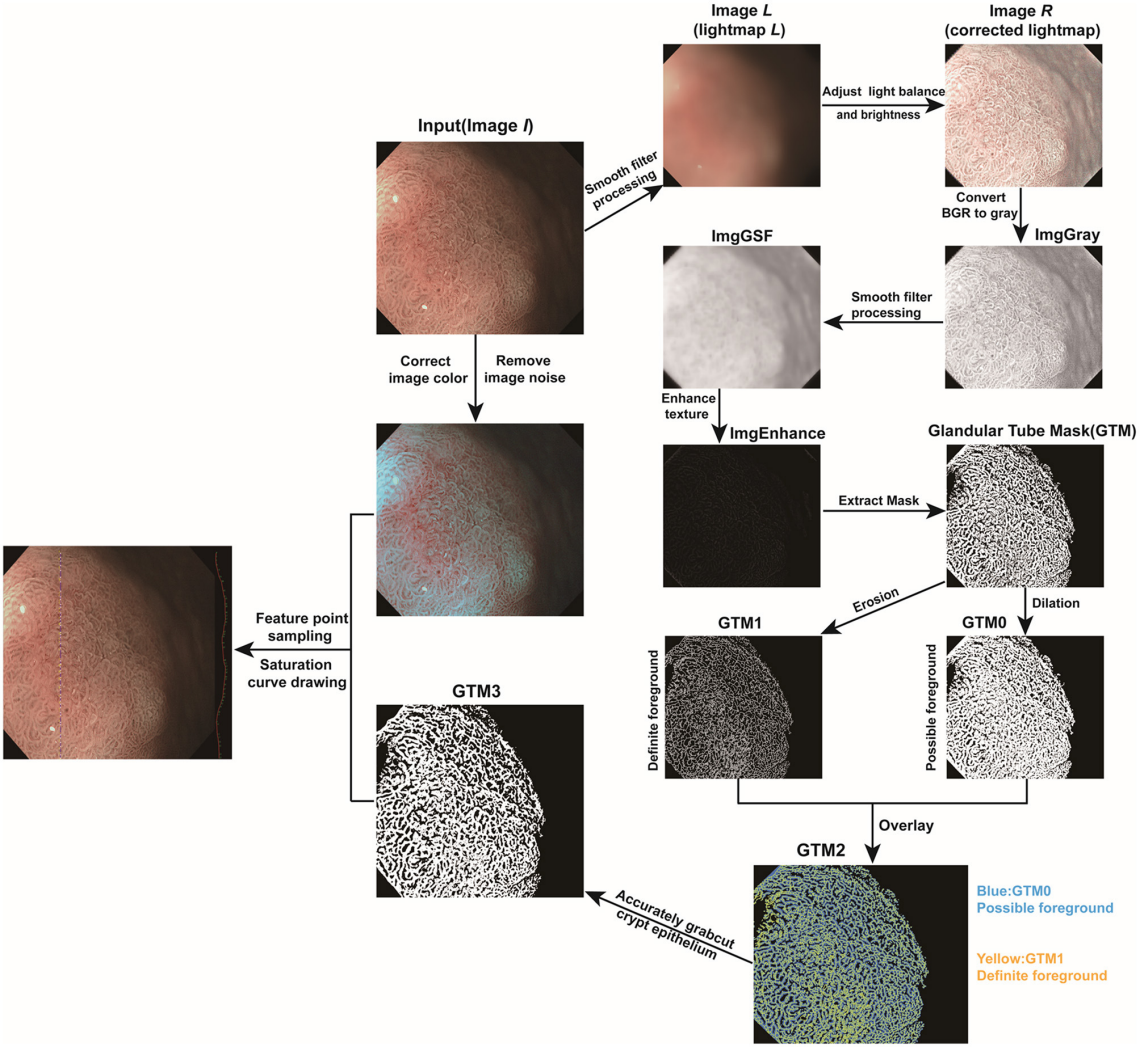
Endoscopic image processing and color feature extraction were performed with Python.

First, a line was drawn artificially in the endoscope image. Then the part of the superficial mucosal epithelium was extracted based on the GTM3. The point with the highest saturation value in each part of the superficial mucosal epithelium were extracted and their saturation values recorded. It is worth noting that the range of the saturation values was 0~255. The greater the saturation value, the closer the color of the superficial mucosal epithelium was to red, whereas the smaller the saturation value, the closer the color of superficial mucosal epithelium was to white. All processing was performed with OpenCV library (18) in Python. The detailed steps of endoscopic image processing are summarized in Figure 4.

### Pathological Image Processing

**Figure 5:**
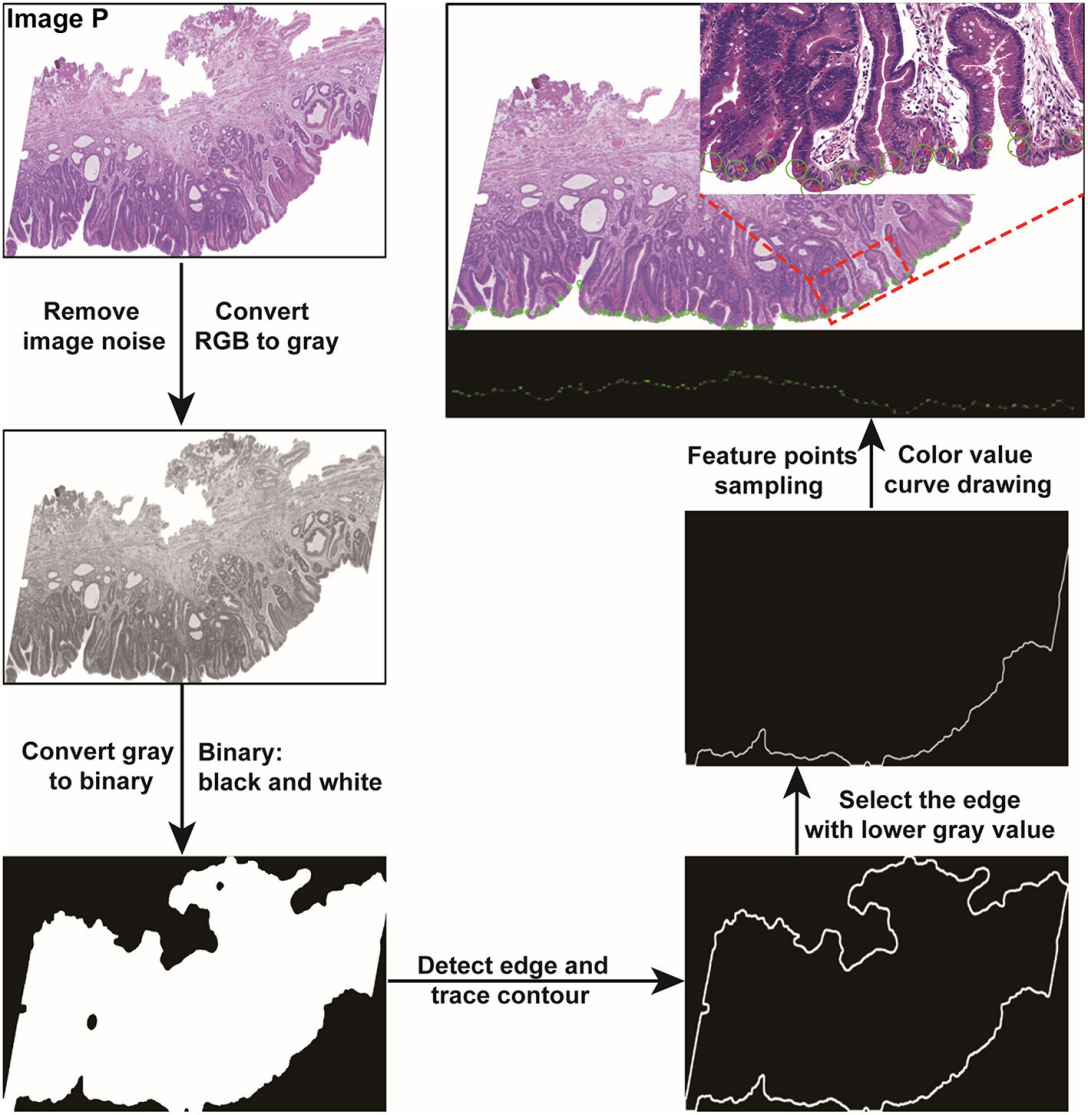
Pathological image processing and color feature extraction were performed with Python.

Given a pathological image P, the Gaussian Blur function (17) was firstly employed to remove the noise. Then the pathological image was converted to a grayscale image. After a simple thresholding operation, we used the Otsu thresholding algorithm (13) to generate the mask of the pathological image. Edge detection in the mask was performed to obtain the edge contour. We then cut the contour into upper and lower parts, calculated the average gray values of the two parts, and took the part with the lower gray value as the edge zone for analysis. All processing was performed with OpenCV library (18) in Python. The detailed pathological image-processing procedure is shown in Figure 5.

### Similarity Evaluation

**Figure 6:**
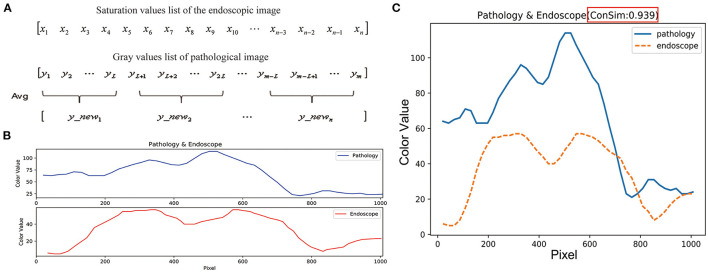
Similarity evaluation of the trends in color curves for endoscopic images and pathological images. **(A)** The length of the endoscopic saturation value list is n, the length of the pathological gray value list is m, and the sampling step length is calculated as: L = m/n. List of gray values for the pathological image is averagely sampled by using the step length of L, and the pathological image gray value list with length n was obtained. **(B)** The length between endoscopic image saturation value list and pathological image gray value list is consistent. **(C)** Cosine values and color curves for the endoscopic and pathological images are summarized in one figure.

The lengths of the lists of color values for the feature points in the pathological and endoscopic images differ because the sizes of the pathological and endoscopic images differ. To calculate the cosine similarity between the saturation values of the endoscopic images and the gray values of the pathological images, the list of gray values for each pathological image was averagely sampled (Figure 6A) to make its length consistent with the length of the list of saturation values for the endoscopic image (Figure 6B).

List of saturation values for an endoscopic image in the line:


$$\begin{array}{l} \left[\begin{array}{ccccccccccccccc} x_{1} & x_{2} & x_{3} & x_{4} & x_{5} & x_{6} & x_{7} & x_{8} & x_{9} & x_{10} & \cdots & x_{n-3} & x_{n-2} & x_{n-1} & x_{n} \end{array}\right] \end{array}$$

The gray values list of a pathological image in the edge zone:


$$\begin{array}{l} \left[\begin{array}{ccccccccccccccc} y_{1} & y_{2} & y_{3} & y_{4} & y_{5} & y_{6} & y_{7} & y_{8} & y_{9} & y_{10} & \cdots & y_{m-3} & y_{m-2} & y_{m-1} & y_{m} \end{array}\right] \end{array}$$

Cosine similarity is a metric used to determine how similar vectors are, irrespective of their size. Mathematically, it calculates the cosine of the angle between two vectors projected in a multidimensional space (19, 20). The formula used is:


$$\text{Similarity}=\text{Cos}\left(\text{θ}\right)\text{=}\frac{\text{x }\cdot \text{ y}}{\left\|\text{x}\right\|\times \left\|\text{y}\right\|}\text{=}\frac{\sum_{\text{i=1}}^{\text{n}}\left({\text{x}}_{\text{i}}\times {\text{y}}_{\text{i}}\right)}{\sqrt{\sum_{\text{i=1}}^{\text{n}}{\left({\text{x}}_{\text{i}}\right)}^{\text{2}}}\times \sqrt{\sum_{\text{i=1}}^{\text{n}}{\left({\text{y}}_{\text{i}}\right)}^{\text{2}}}}$$

When the two vectors have the same direction, the cosine similarity value is 1; when the directions of the two vectors are opposite, the cosine similarity value is−1; and when the angle between the two vectors is 90°, the cosine similarity value is 0 (19, 20). Thus, the cosine similarity value ranges from−1 to 1, and the closer the value is to 1, the closer are the directions of the two lists, i.e., the more similar are the two lists (Figure 6C). The cosine similarity was also calculated with Python.

### Participating Endoscopists

We recruited as study participants 12 endoscopists from our department who had previously used the VS classification system for EGC diagnosis. Six of them were assigned to the “‘experienced” group, because they had performed more than 10,000 conventional endoscopy examinations, and the other six were assigned to the “junior” group, because they had performed fewer than 10,000 but more than 2,000 conventional endoscopic examinations. The participant demographics are presented in Table 1. To minimize the diagnostic variation between these endoscopists, they were trained in using the pink pattern to diagnose gastric cancer with ME-NBI. It took 2 days to train 12 endoscopists (including both junior and experienced) to recognize the pink pattern, and all of the endoscopists fullfilled the criteria. How the endoscopists were trained to use the pink pattern is described in the Supplementary Material.

**Table 1:** Characteristics of the 12 participants.

**Characteristic**	**Value**
Sex, *n*	
Male	7
Female	5
Mean age, years(SD)	34.3 (4.7)
No. of endoscopies conducted, *n*	
<10,000 (junior)	6
≥10,000 (experienced)	6
No. of cases for differential diagnosis by using ME-NBI, *n*	
<100	4
≥100	8

*SD, Standard deviation; ME-NBI, magnifying endoscopy with narrow-band imaging*.

### Diagnostic Tests

ME-NBI images of 185 gastric lesions were selected for diagnostic testing (95 cancerous and 90 non-cancerous). The characteristics of the 185 lesions are shown in Table 2. All of the images from these 185 lesions were taken by an expert endoscopist at Zhejiang Cancer Hospital, Hangzhou, China. Two diagnostic tests were undertaken in this study, on the same 185 gastric lesions. The time gap between performance of test 1 and test 2 is 2 months. Endoscopists were randomized to perform test 1 or test 2 first by drawing lots. In order to reduce the impact of fatigue on diagnosis, each endoscopist went through 95 images and took a 1 h break in the two tests. After the break, remaining 90 images were continued to be evaluated. All images were randomly presented by computer in both tests. In test 1, each participant evaluated the ME-NBI images according to the VS classification system; in test 2, the images of these 185 gastric lesions were evaluated by these participants based on the VS classification plus the pink pattern. The 12 endoscopists were isolated and performed the two tests alone, to avoid interference by the other participants. A detailed flow chart of the diagnostic tests is shown in Supplementary Figure 4.

**Table 2:** Characteristics of the 185 gastric lesions used for diagnostic tests.

**Characteristics**	**Cancer**	**Non-cancer**
	**(*n* = 95)**	**(*n* = 90)**
Histologic type, *n* (%)		
Well-differentiated adenocarcinoma	69 (72.6)	NA
Moderately differentiated adenocarcinoma	26 (27.4)	NA
Low-grade adenoma	NA	7 (7.8)
Chronic gastritis	NA	83 (92.2)
Macroscopic appearance, *n* (%)		
Elevated lesion	29 (30.5)	17 (18.9)
Flat lesion	11 (11.6)	24 (26.7)
Depressed lesion	55 (57.9)	49 (51.6)
Mean size, *n* (%)		
≥1cm	67 (70.5)	19 (21.1)
<1 cm	28 (29.5)	71 (78.9)
Depth of invasion, *n* (%)		
Mucosa	83 (87.4)	90 (100)
Submucosa	12 (12.6)	0
Location, *n* (%)		
Lower third	33 (34.7)	39 (43.3)
Middle third	39 (41.1)	35 (38.9)
Upper third	23 (24.2)	16 (17.8)

*NA, Not applicable*.

### Statistical Analysis

The diagnostic performances of test 1 and test 2 were evaluated by calculating the AUC, accuracy, sensitivity, specificity, positive predictive value (PPV), and negative predictive value (NPV). The primary outcome of the study was the changes in diagnostic accuracy between test 1 and test 2. The second outcome in this study were the sensitivity, specificity, PPV and NPV when used VS classification system and pink pattern to diagnose EGC. The differences in these diagnostic values between tests 1 and 2 were calculated with the Mann–Whitney *U* test. The agreement of the 12 endoscopists was evaluated with Fleiss' kappa value (slight agreement: 0.01–0.20; fair agreement: 0.21–0.40; moderate agreement: 0.41–0.60; substantial agreement: 0.61–0.80, and almost perfect agreement: 0.81–1.00) (21). Each test was two-sided, and *P* < 0.05 was considered statistically significant. All analyses were conducted with R, version 4.0.3 (R Project for Statistical Computing, Vienna, Austria) and GraphPad Prism 8 (GraphPad Software, Inc., San Diego, CA).

## Results

### Cosine Similarity Between the Endoscopic and Pathological Images

We used the computer image-processing technology described in the Methods section to extract the color features of selected high quality endoscopic images and pathological images of 20 representative lesions, and then calculated the cosine similarity between the endoscopic images and the pathological images of the same lesions. The results showed that the cosine similarity between the color values of the ME-NBI images and HE-stained pathological images of 20 lesions were all > 0.70 (median, 0.894; minimum 0.744; maximum, 0.981), indicating that the pink pattern visualized in the ME-NBI images correlated strongly with the color changes in the HE-stained pathological images of these 20 lesions. The detailed cosine similarity values and other information are summarized in Table 3.

**Table 3:** The detailed information of lesions selected for image color extraction to calculate cosine similarity.

**Lesion No**.	**Sex**	**Age (years)**	**Location**	**Lesion size (cm)**	**Invasion depth**	**Histological type**	**Paris classification**	**ConSim**
1	Female	53	Gastric antrum	1.0	Mucosa	Well differentiated	IIb	0.939
2	Female	53	Gastric angle	2.5	Mucosa	Well differentiated	IIa	0.833
3	Male	66	Gastric angle	1.0	Mucosa	Well differentiated	IIa+IIc	0.936
4	Male	82	Gastric antrum	1.0	Mucosa	Moderately to well differentiated	IIa	0.892
5	Female	58	Gastric angle	1.0	Mucosa	Moderately to well differentiated	IIc	0.882
6	Male	66	Cardia	2.5	Mucosa	Well differentiated	IIb	0.948
7	Male	64	Gastric antrum	2.4	Mucosa	Moderately differentiated	IIa+IIc	0.84
8	Male	64	Gastric antrum	1.2	Mucosa	Moderately differentiated	IIc+IIb	0.856
9	Male	64	Junction of gastric	2.4	Mucosa	Well differentiated	IIb	0.936
			antrum and body					
10	Male	45	Gastric antrum	4.0	Mucosa	Well differentiated	IIa+IIc	0.857
11	Male	66	Gastric angle	1.5	Mucosa	Moderately differentiated	IIc	0.895
12	Male	62	Gastric antrum	1.2	Mucosa	Poorly to moderately differentiated	IIc	0.831
13	Male	58	Gastric body	1.2	Mucosa	Well differentiated	IIa+IIc	0.907
14	Male	58	Gastric body	1.0	Mucosa	Well differentiated	IIb	0.892
15	Female	63	Gastric antrum	1.0	Mucosa	Well differentiated	IIb	0.833
16	Male	77	Lower edge of cardia	2.0	Mucosa	Poorly to moderately differentiated	IIb	0.896
17	Female	56	Cardia	1.0	Mucosa	Moderately to well differentiated	IIc	0.96
18	Male	73	Cardia	1.0	Mucosa	Moderately to well differentiated	IIa	0.93
19	Male	58	Gastric antrum	2.0	Mucosa	Well differentiated	IIa	0.744
20	Female	61	Lower edge of cardia	2.0	Mucosa	Poorly to moderately differentiated	IIc	0.981

*ConSim, Cosine similarity*.

### Comparison of the Diagnostic Performances in Tests 1 and 2

**Figure 7:**
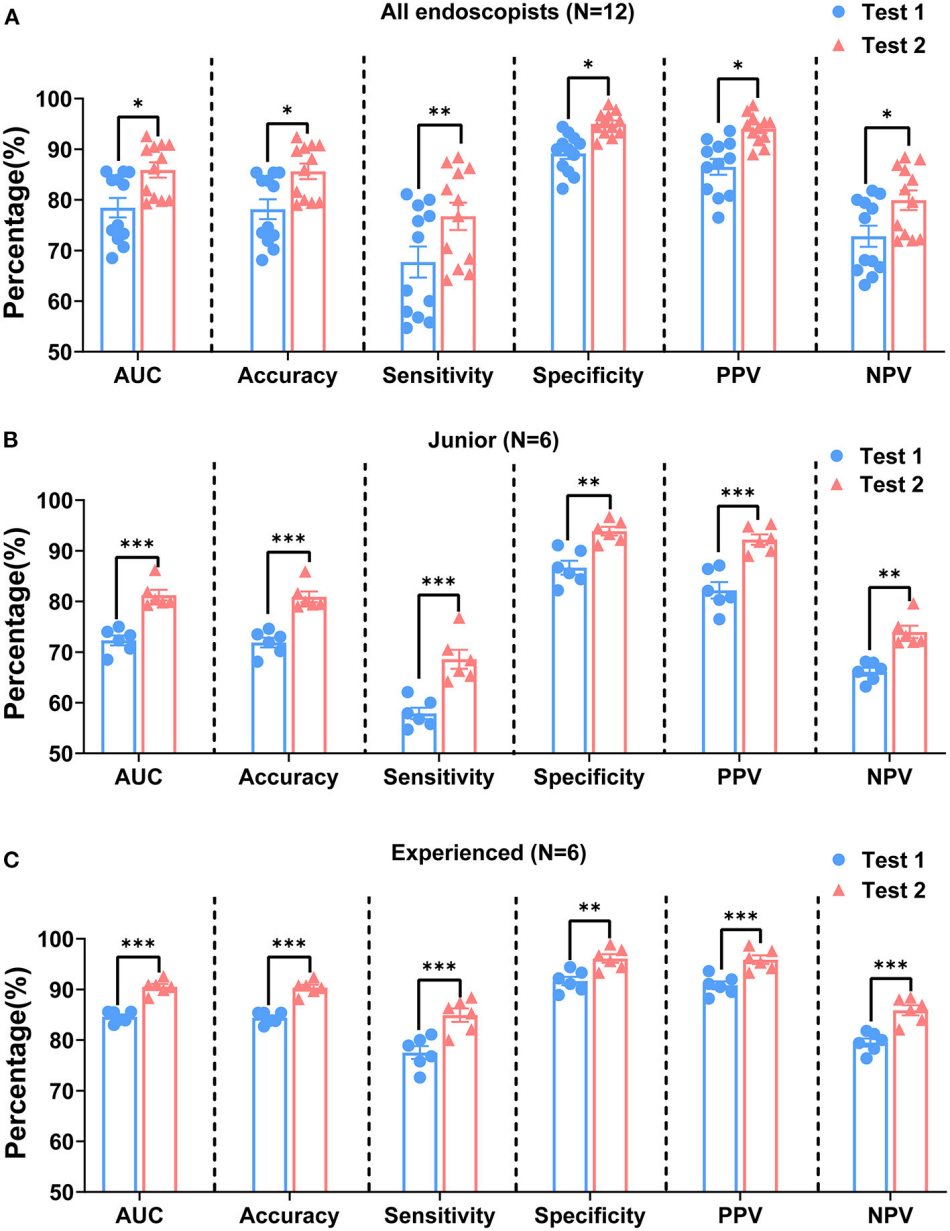
Comparison of diagnostic performances of tests 1 and 2 by all endoscopists **(A)**, junior group **(B)**, and experienced group **(C)**. AUC, area under curve; PPV, positive predictive value; NPV, negative predictive value. *P*-values were calculated with the Mann–Whitney *U* test. ^*^P < 0.05, ^**^P < 0.01, and ^***^P < 0.001.

In test 1, the endoscopists evaluated the ME-NBI images according to the VS classification system, whereas in test 2, all the participants made these endoscopic diagnoses based on the VS classification system plus the pink pattern. When we assessed the overall diagnostic performance, the median AUC, accuracy, and specificity of test 1 were 79.0, 78.7, and 90.0% for all endoscopists, respectively, whereas sensitivity was only 67.4% (Table 4). However, in test 2, the median values for AUC, accuracy, sensitivity, and specificity increased to 87.3, 87.0, 78.4, and 95.0%, respectively, meaning that the diagnostic capability of ME-NBI for cancer was significantly improved when the pink pattern was taken into account on the ME-NBI diagnosis (Figure 7A). In junior and experienced subgroups, the AUC, accuracy, sensitivity and specificity for test 2 were significantly higher than those for test 1 (Figures 7B,C). PPV and NPV were also significantly better in test 2 than in test 1 in the group of all endoscopists and the junior and experienced groups (Table 4, Figure 7). Interestingly, in test 2, although specificity did not differ significantly between the experienced and junior groups, it still tended to be greater in the experienced group, and AUC, accuracy, sensitivity, PPV and NPV were all significantly better in the experienced group than in the junior group (Supplementary Figure 5).

**Table 4:** Diagnostic performance comparison between tests 1 and 2 for all gastric lesions.

**Diagnostic parameter**	**All endoscopists (*n* = 12)**		**Junior (*n* = 6)**		**Experienced (*n* = 6)**	
	**Test 1**	**Test 2**	**Test 1**	**Test 2**	**Test 1**	**Test 2**
AUC (%), median (range)	79.0 (68.5–85.6)	87.3 (79.3–92.6)	72.8 (68.5–75.0)	80.2 (79.3–86.2)	84.8 (83.0–85.6)	90.7 (88.3–92.6)
Accuracy (%), median (range)	78.7 (68.1–85.4)	87.0 (79.0–92.4)	72.5 (68.1–74.6)	90.6 (88.1–92.4)	84.6 (82.7–85.4)	90.6 (88.1–92.4)
Sensitivity (%), median (range)	67.4 (54.7–81.1)	78.4 (64.2–88.4)	57.4 (54.7–62.1)	67.4 (64.2–76.8)	77.9 (82.6–81.1)	85.8 (80.0–88.4)
Specificity (%), median (range)	90.0 (82.2–94.4)	95.0 (91.1–98.9)	86.2 (82.2–91.1)	93.9 (91.1–96.7)	91.7 (88.9–94.4)	96.2 (93.3–98.9)
PPV (%), median (range)	87.7 (76.5–93.6)	94.5 (89.0–98.7)	81.5 (76.5–87.1)	92.2 (89–95.3)	90.8 (88.2–93.6)	95.8 (93.3–98.7)
NPV (%), median (range)	72.25 (63.2–81.8)	80.9 (71.9–88.4)	66.4 (63.2–68.1)	72.7 (71.9–79.6)	79.7 (76.4–81.8)	86.4 (82.1–88.4)

*AUC, area under curve; PPV, positive predictive value; NPV, negative predictive value*.

### Interobserver Agreement

Table 5 shows the interobserver agreement among the 12 participants in tests 1 and 2. Fleiss' kappa values for all the endoscopists, for the junior group, and for experienced groups increased from test 1 to test 2 (from 0.51 to 0.65, from 0.47 to 0.58, and from 0.60 to 0.76, respectively). According to this analysis, although experience in performing endoscopy differed among these participants, moderate agreement on test 1 was demonstrated among all the endoscopists and within the junior and experienced groups. However, the agreement between all the endoscopists and within the experienced group improved substantially on test 2.

**Table 5:** Fleiss' kappa value of tests 1 and 2 in 12 endoscopists.

**Endoscopists**	**Test 1**		**Test 2**	
	**Fleiss' kappa (95%CI)**	**Agreement**	**Fleiss' kappa (95%CI)**	**Agreement**
All (*n* = 12)	0.51 (0.49–0.52)	Moderate	0.65 (0.63–0.67)	Substantial
Junior (*n* = 6)	0.47 (0.43–0.51)	Moderate	0.58 (0.54–0.62)	Moderate
Experienced (*n* = 6)	0.60 (0.56–0.64)	Moderate	0.76 (0.73–0.80)	Substantial

*Fleiss' kappa values were evaluated by R with raters package. Kappa values of 0.01 to 0.20, 0.21 to 0.40, 0.41 to 0.60, 0.61 to 0.80, and 0.81 to 1.00 were defined as slight agreement, fair agreement, moderate agreement, substantial agreement, and almost perfect agreement, respectively*.

## Discussion

In the diagnosis of gastric mucosal lesions with endoscopy, it is important to distinguish between tumors and non-tumors. Pathological evaluation by biopsy is the gold standard for the diagnosis of gastric epithelial tumors. Most endoscopists especially the inexperienced usually take multiple biopsies for one patient during the procedure because they are not confident for their diagnoses, which aggravates tissue damage. Therefore, it is imperative to develop a method to minimize the number of biopsies taken during endoscopy. Studies have confirmed that ME-NBI significantly improved the diagnostic performance for gastric mucosal lesions and reduced the number of biopsies required when diagnosing tumors (10). At present, the VS classification system is commonly used to diagnose gastric cancer under ME-NBI. When the VS classification system is used to determine whether a suspicious lesion is cancerous, the DL must first be identified (8). However, not all suspicious lesions have a clear DL and/or IMVP or IMSP (5, 10). When the DL and/or IMVP or IMSP are indeterminate, the accuracy of the VS classification system in diagnosing gastric cancer decreases. Under these circumstances, the diagnosis depends predominantly on lesion biopsies. How can we maintain a high accuracy rate in diagnosing gastric cancer without increasing the number of biopsies when neither the DL nor IMVP/IMSP can be determined?

Cancerous lesions are often accompanied by vascular changes. Abnormal blood vessels can also show reddish-brown color changes under ME-NBI. Therefore, when the pink pattern appears in the cancerous area, it is easily confused with the color change associated with abnormal blood vessels in the same area, so the pink pattern is overlooked on ME-NBI examination. Therefore, any vascular interference must be removed to allow the recognition of the pink pattern in suspicious lesions under ME-NBI. Additionally, pathological diagnoses demonstrated that the nucleus-to-cytoplasm ratio of the gastric epithelial cells in the cancerous area showed the pink pattern under ME-NBI was increased. Then we speculated that the pink pattern in the cancerous area under ME-NBI is related to the change in the nucleus-to-cytoplasm ratio of gastric epithelial cells in the same area. However, the mechanism linking the pink pattern with the nucleus-to-cytoplasm ratio of gastric epithelial cells was still unknown.

NBI has two light bands with central wavelengths of 415 nm (blue light) and 540 nm (green light) (22). The dual-wavelength light band is confined to the mucosa and does not penetrate the submucosal tissue (22), so the microstructure of the mucosal surface can be clearly visualized. Based on these characteristics of the NBI light band, we tried to identify the linkage between the pink pattern and the nucleus-to-cytoplasm ratio by analyzing the color features of the crypt epithelium on the mucosal surface in NBI images and the epithelial cell layer in pathological images. In the pathological images, the nuclei look dark, whereas the cytoplasm is lighter with HE staining. The nucleus-to-cytoplasm ratio increases when the epithelial cells become cancerous, and the color of the cancerous area becomes correspondingly darker with HE staining. Hence, the change in the nucleus-to-cytoplasm ratio of epithelial cells is reflected in the color change in these cells. The color change trend in the cancerous epithelial cells in HE-stained pathological images was consistent with the color change trend in the cancerous lesions in the ME-NBI images, confirming that the increased nucleus-to-cytoplasm ratio of gastric cancer epithelial cells correlates strongly with the pink pattern in cancerous lesions on ME-NBI images. Therefore, this color change is a key factor linking the pink pattern to the nuclear-to-cytoplasm ratio of cancerous epithelial cells.

To verify that the pink pattern of the cancerous lesions on ME-NBI images correlated strongly with the increase in the nucleus-to-cytoplasm ratio of gastric cancer epithelial cells, the color features of ME-NBI images and HE-stained pathological images of the cancerous gastric mucosal surface were extracted and quantified. The result of cosine similarity analysis by using the extracted color features showed that the pink color change in the cancerous lesions on ME-NBI images was consistent with the color change in the cancerous epithelial cells in HE-stained pathological images, suggesting that the presence of a pink pattern is a warning sign, and can be part of a preoperative assessment strategy for early gastric cancer in clinical practice.

Stored images captured endoscopically with NBI were evaluated independently by 12 endoscopists using the VS classification system (test 1) and the VS classification system plus the pink pattern (test 2) to assess the clinical usefulness of the pink pattern under ME-NBI. When performed by all endoscopists, test 2 achieved significantly higher AUC, accuracy, sensitivity, specificity, PPV, and NPV than test 1.When the two tests were compared specifically within the junior or experienced group, test 2 again showed significantly better AUC, accuracy, sensitivity, specificity, PPV, and NPV. Very importantly in clinical practice, tests with high PPV and NPV allow endoscopists to identify the lesion that require a pathological diagnosis, which may potentially allow the so-called “optical biopsy” to be realized. Next, we calculated Fleiss' kappa value for all endoscopists and for the junior and experienced groups in test 1 and test 2 to evaluate the agreement in endoscopic diagnoses among the participants. The kappa values for all the endoscopists and the junior and experienced groups in test 1 and test 2 indicated at least moderate agreement, and the agreement among all endoscopists and within the experienced group improved substantially in test 2. The moderate or higher diagnostic agreement among the participants is mainly attributable to the fact that the endoscopists were all from the same department and used a similar diagnostic algorithm, which guaranteed the consistency of the test.

**Figure 8:**
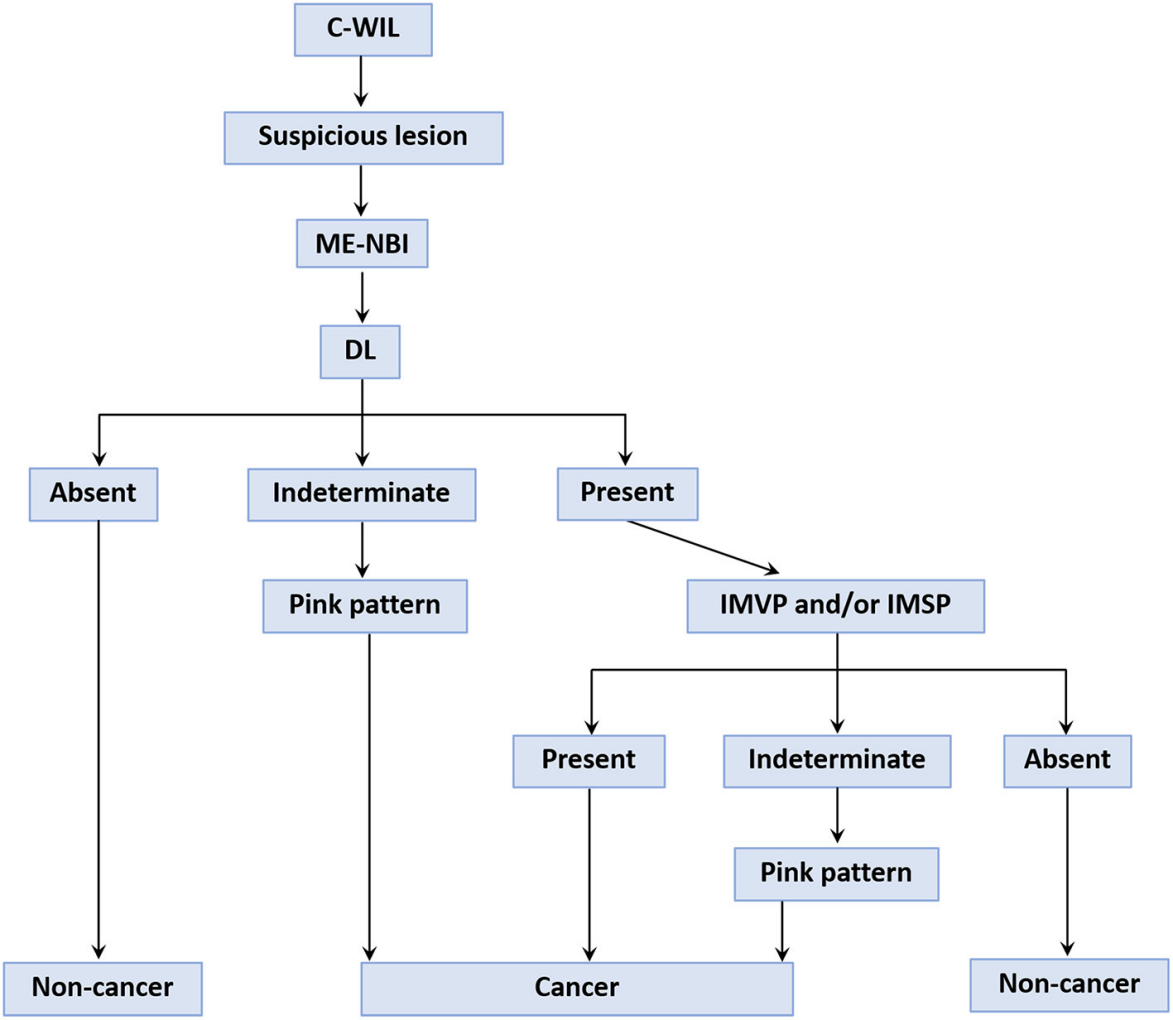
A provisional strategy for gastric cancer diagnosis using the “pink pattern” to supplement to the VS classification system. C-WIL, conventional white light imaging; ME-NBI, magnifying endoscopy with narrow-band imaging; DL, demarcation line; IMVP, irregular microvascular pattern; IMSP, irregular microsurface pattern.

From the results of this study, we devised a provisional strategy for the diagnosis of differentiated EGC under ME-NBI by using the pink pattern to supplement the VS classification system (Figure 8). Briefly, a gastric endoscopic examination is first performed with conventional white-light imaging. When a suspicious lesion is detected, the endoscope should be switched to ME-NBI to differentiate cancer from non-cancer. The key step in distinguishing gastric cancer and non-cancer is to determine the DL between the suspicious lesion and the background mucosa. If a DL is absent, it is easy to diagnose the suspicious lesion as non-cancerous. If the DL is indeterminate, the pink pattern can be taken into account and a diagnosis of a cancerous lesion can probably be made. If a DL is clearly visible, the subsequent presence of IMVP and/or IMSP should be determined. If IMVP and/or IMSP are identified within the DL, the suspicious lesion can be diagnosed as gastric cancer. If IMVP and/or IMSP are absent, the lesion is identified as non-cancerous. However, if IMVP and/or IMSP are indeterminate within the DL, the pink pattern will also be helpful in the diagnosis of EGC. Our study had several limitations. First, it was a retrospective study and did not involve real-time assessment; all endoscopic diagnoses were made from stored images. Selection bias may have occurred when the stored images were selected for the two diagnostic tests. Second, we recruited the 12 endoscopists to perform diagnostic tests from only one institution, which may have led to participant bias. Third, the numbers of lesions and endoscopists in our study were small, so the power to evaluate diagnostic performance may have been insufficient. To overcome these limitations, we will conduct an on-site real-time evaluation of newly detected and undiagnosed gastric lesions in a future study. Larger numbers of patients and endoscopists from multiple centers will also be included in the future study to evaluate the diagnostic performance of the pink pattern for EGC.

In conclusion, we found that cancerous gastric lesions showed a pink color change under ME-NBI, and designated this phenomenon the “pink pattern”. We then identified a strong correlation between the pink pattern on ME-NBI images and the change in the nucleus-to-cytoplasm ratio in gastric epithelial cells. The results of this study demonstrated that the pink pattern detected under ME-NBI was an extremely useful marker for the diagnosis of differentiated EGC. We also proposed a provisional strategy for the diagnosis of differentiated EGC under ME-NBI using the pink pattern to supplement the VS classification system.

## Data Availability Statement

The raw data supporting the conclusions of this article will be made available by the authors, without undue reservation.

## Ethics Statement

The studies involving human participants were reviewed and approved by Ethics Committee of the Zhejiang Cancer Hospital. The patients/participants provided their written informed consent to participate in this study.

## Author Contributions

SW and SC designed the study. JY, RR, and YL contributed to the data collection. CS, HW, JJ, MC, and CJ did the images processing and cosine similarity calculation. YT and ZC did the data analysis, under supervision of SW and SC. SC, JY, and RR drafted the paper and interpreted the results. SC, HW, and SW revised the manuscript. All authors contributed to the article and approved the submitted version.

## Conflict of Interest

CS, HW, JJ, MC, and CJ were employed by the company Hithink RoyalFlush Information Network Co., Ltd. The remaining authors declare that the research was conducted in the absence of any commercial or financial relationships that could be construed as a potential conflict of interest.

## Publisher's Note

All claims expressed in this article are solely those of the authors and do not necessarily represent those of their affiliated organizations, or those of the publisher, the editors and the reviewers. Any product that may be evaluated in this article, or claim that may be made by its manufacturer, is not guaranteed or endorsed by the publisher.

## Abbreviations

ME-NBI: magnifying endoscopy with narrow-band imaging
EGC: early gastric cancer
AUC: area under curve
PPV: positive predictive value
NPV: negative predictive value
VS: vessel-plus-surface
DL: demarcation line
IMVP: irregular microvascular pattern
IMSP: irregular microsurface pattern
ESD: Endoscopic submucosal dissection
C-WIL: white-light imaging.

## References

[1] Sung H Ferlay J Siegel RL Laversanne M Soerjomataram I Jemal A. Global cancer statistics 2020: GLOBOCAN estimates of incidence and mortality worldwide for 36 cancers in 185 countries. CA Cancer J Clin. (2021) 0:1–41. 10.3322/caac.21660 33538338

[2] Kaltenbach T Sano Y Friedland S Soetikno R. American gastroenterological association (AGA) institute technology assessment on image-enhanced endoscopy. Gastroenterology. (2008) 134:327–40. 10.1053/j.gastro.2007.10.062 18061178

[3] Muto M Katada C Sano Y Yoshida S. Narrow band imaging: a new diagnostic approach to visualize angiogenesis in superficial neoplasia. Clin Gastroenterol Hepatol. (2005) 3:S16–20. 10.1016/S1542-3565(05)00262-4 16012987

[4] Yao K Anagnostopoulos GK Ragunath K. Magnifying endoscopy for diagnosing and delineating early gastric cancer. Endoscopy. (2009) 41:462. 10.1055/s-0029-1214594 19418401

[5] Ezoe Y Muto M Uedo N Doyama H Yao K Oda I. Magnifying narrowband imaging is more accurate than conventional white-light imaging in diagnosis of gastric mucosal cancer. Gastroenterology. (2011) 141:2017–25. 10.1053/j.gastro.2011.08.007 21856268

[6] Uedo N Ishihara R Iishi H Yamamoto S Yamada T Imanaka K. A new method of diagnosing gastric intestinal metaplasia: narrow-band imaging with magnifying endoscopy. Endoscopy. (2006) 38:819. 10.1055/s-2006-944632 17001572

[7] Yao K Oishi T Matsui T Yao T Iwashita A. Novel magnified endoscopic findings of microvascular architecture in intramucosal gastric cancer. Gastrointest Endosc. (2002) 56:279–84. 10.1067/mge.2002.126061 12145613

[8] Muto M Yao K Kaise M Kato M Uedo N Yagi K. Magnifying endoscopy simple diagnostic algorithm for early gastric cancer (MESDA-G). Digest Endosc. (2016) 28:379–93. 10.1111/den.12638 26896760

[9] Doyama H Nakanishi H Yao K. Image-enhanced endoscopy and its corresponding histopathology in the stomach. Gut Liver. (2020) 15:329–37. 10.5009/gnl19392 32200589 PMC8129655

[10] Yao K Doyama H Gotoda T Ishikawa H Nagahama T Yokoi C. Diagnostic performance and limitations of magnifying narrow-band imaging in screening endoscopy of early gastric cancer: a prospective multicenter feasibility study. Gastric Cancer. (2014) 17:669–79. 10.1007/s10120-013-0332-0 24407989

[11] Schlemper RJ Riddell RH Kato Y Borchard F Cooper HS Dawsey SM. The Vienna classification of gastrointestinal epithelial neoplasia. Gut. (2000) 47:251–5. 10.1136/gut.47.2.251 10896917 PMC1728018

[12] Min D Choi S Lu J Ham B Sohn K Do MN. Fast global image smoothing based on weighted least squares. IEEE Trans Image Process. (2014) 23:5638–53. 10.1109/TIP.2014.2366600 25373085

[13] Zhu N Wang G Yang G Dai W. A Fast 2D Otsu Thresholding algorithm based on improved histogram. In: Proceedings of 2009 Chinese Conference on Pattern Recognition. Nanjing (2009). p. 1–5.

[14] Wei S Kai Z Yuan J Yan W Xiang B Yuille A. DeepSkeleton: learning multi-task scale-associated deep side outputs for object skeleton extraction in natural images. IEEE Trans Image Process. (2017) 26:5298–311. 10.1109/TIP.2017.2735182 28783632

[15] Tao W. Iterative narrowband-based graph cuts optimization for geodesic active contours with region forces (GACWRF). IEEE Trans Image Process. (2012) 21:284–96. 10.1109/TIP.2011.2160955 21724512

[16] Guanghua C Xiaolong Z. A Method to improve robustness of the gray world algorithm. In: Proceedings of 4th International Conference on Computer, Mechatronics, Control and Electronic Engineering. Hangzhou (2015). p. 250–5.

[17] Gedraite ES Hadad M. Investigation on the effect of a Gaussian blur in image filtering and segmentation. In: Proceedings of ELMAR-2011. Zadar (2011). p. 393–6.

[18] Bradski G Kaehler AO. Dr. Dobb's journal of software tools. San Francisco: UBM Technology Group (2000). p. 3.

[19] Novotný V. Implementation notes for the soft cosine measure. In: CIKM '18: Proceedings of the 27th ACM International Conference on Information and Knowledge Management. (2018). 10.1145/3269206.3269317

[20] Sidorov G Gelbukh A Gómez-Adorno H Pinto D. Soft similarity and soft cosine measure: similarity of features in vector space model. Computación y Sistemas. (2014) 18:491–504. 10.13053/cys-18-3-2043

[21] Ikehara H Doyama H Nakanishi H Hatta W Gotoda T Ishikawa H. Analysis of factors related to poor outcome after e-learning training in endoscopic diagnosis of early gastric cancer using magnifying narrow-band imaging. Gastrointest Endosc. (2019) 90:440–7. 10.1016/j.gie.2019.04.230 31034809

[22] Yao K Takaki Y Matsui T Iwashita A Anagnostopoulos GK Kaye P. Clinical application of magnification endoscopy and narrow-band imaging in the upper gastrointestinal tract: new imaging techniques for detecting and characterizing gastrointestinal Neoplasia. Gastrointest Endosc Clin N Am. (2008) 18:415–33. 10.1016/j.giec.2008.05.011 18674694

