# Supplementary material for: “Pink Pattern” Visualized in Magnifying Endoscopy With Narrow-Band Imaging Is a Novel Feature of Early Differentiated Gastric Cancer: A Bridge Between Endoscopic Images and Histopathological Changes
Source: Front Med (Lausanne). 2021 Nov 15;8:763675. doi: 10.3389/fmed.2021.763675 (PMC8634361; doi:10.3389/fmed.2021.763675)
Supplement: Supplementary file 1 [file Data_Sheet_1.docx]

**The “pink pattern” visualized in magnifying endoscopy with narrow-band imaging is a new feature of early gastric differentiated cancer——a bridge between endoscopic image and histopathological change**

Shengsen Chen^†1^, Jiangping Yu^†1^, Rongwei Ruan^†1^, Yandong Li^†1^, Yali Tao^1^, Qiwen Shen^1^, Zhao Cui^1^, Cheng Shen^2^, Huogen Wang^2^, Jiayan Jin^2^, Ming Chen^2^, Chaohui Jin^2^, Shi Wang*^1^

^1^Department of Endoscopy, Cancer Hospital of the University of Chinese Academy of Sciences(Zhejiang Cancer Hospital), Institute of Cancer and Basic Medicine(IBMC), Chinese Academy of Sciences, Hangzhou, China.

^2^Hithink RoyalFlush Information Network Co., Ltd, Hangzhou, China

^†^These authors contributed to this work equally

*Corresponding author: Shi Wang. Department of Endoscopy, Cancer Hospital of the University of Chinese Academy of Sciences(Zhejiang Cancer Hospital), Institute of Cancer and Basic Medicine(IBMC), Chinese Academy of Sciences, Hangzhou 310022, Zhejiang, China.

E-mail: wangshi@zjcc.org.cn, telephone number: +86-571-88122277, fax number: +86-571-88122277.

**Running title: A “pink pattern” under ME-NBI**

**Supplementary method**

We selected the typical NBI images including the pink pattern, and then take a lecture (first day) for the endoscopists who enrolled in this study. After the lecture (second day), these endoscopists need to complete a quiz about the pink pattern. The quiz has total 100 images (including 50 images has pink pattern), endoscopists who can accurately recognize more than 80% pink pattern were considered as training completion.

**Figure legends**

**Figure S1.** Representative case of “pink pattern” when the demarcation line (DL) is clear. The suspected lesion of early cancer in the lower edge of cardia (the dotted box area). Part(A) is conventional white light imaging findings and part(B) is narrow band imaging findings. (C) Magnified image of the dotted box area in part(A) and (B). The “pink pattern” is observed in the lesion(within the yellow dotted line). The red dotted line indicates DL. The pink color change is easily interfered by the abnormal superficial microvessels when DL is clear.

**Figure S2.** Histopathological findings of FigureS1 under different magnification. (A-C) Hematoxylin and eosin staining of the resected specimen revealed that the histopathological diagnosis was gastric cancer. The color of gastric epithelial cells at the left of red line looks much darker than that at the right of red line. (B) Magnified image of the dotted box area in part (A). (C) Magnified image of the dotted box area in part (B). The nucleus-to-plasma ratio of epithelial cells at the left of red line is increased compared with that at the right of red line.

**Figure S3.** Additional endoscopic images depicting the "pink pattern".

**Figure S4.** Flow chart of the diagnostic test.

**Figure S5.** Diagnostic performance comparison between junior and experienced groups in tests 1 and 2. AUC, area under curve; PPV, positive predictive value; NPV, negative predictive value. P values were calculated by using Mann-Whitney test. *P < 0.05, **P < 0.01, and ***P < 0.001; NS, no significance.


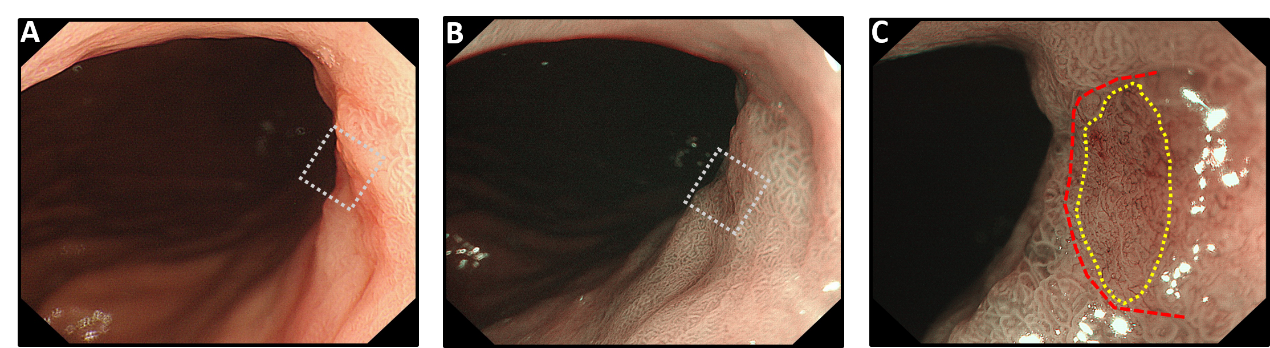


**Figure S1**


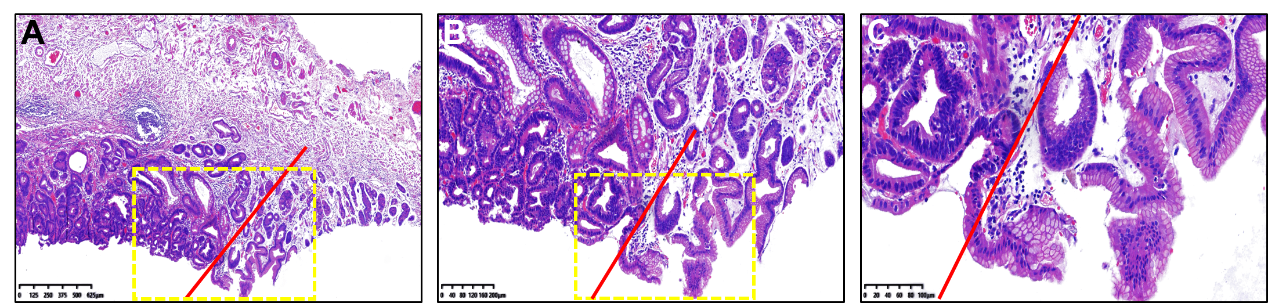


**Figure S2**


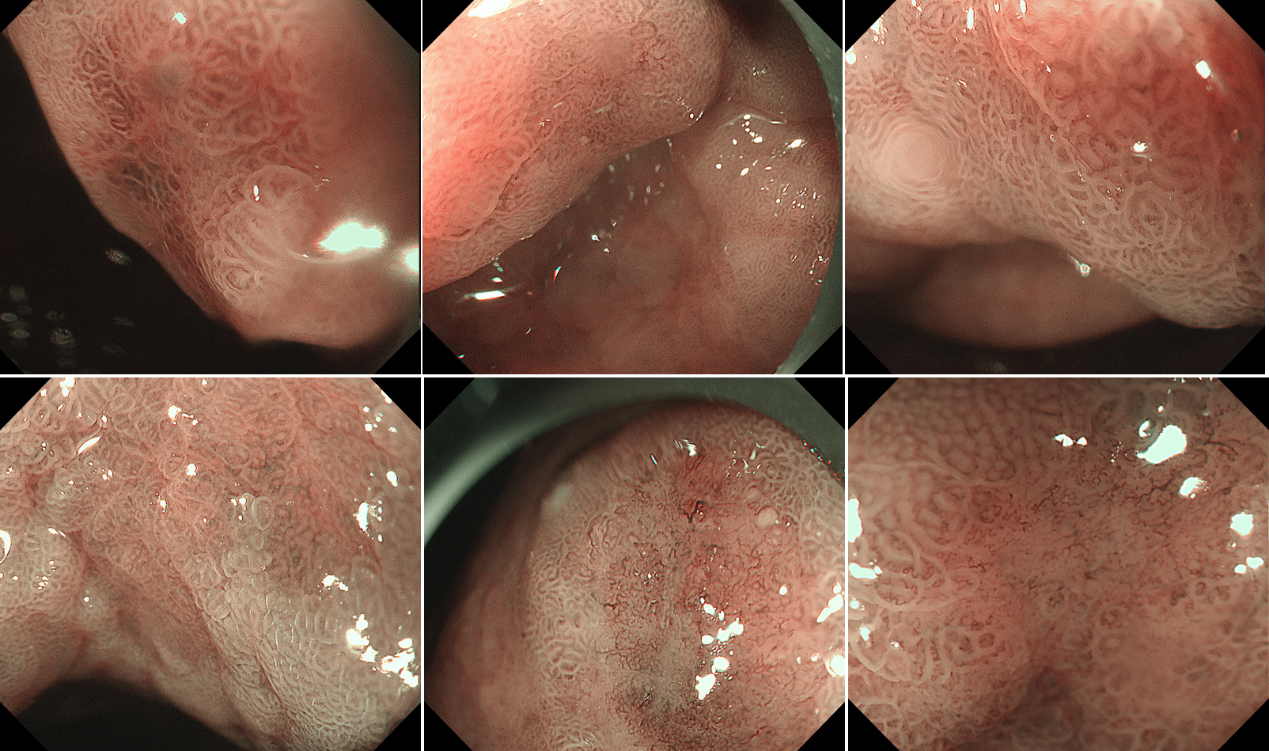


**Figure S3**


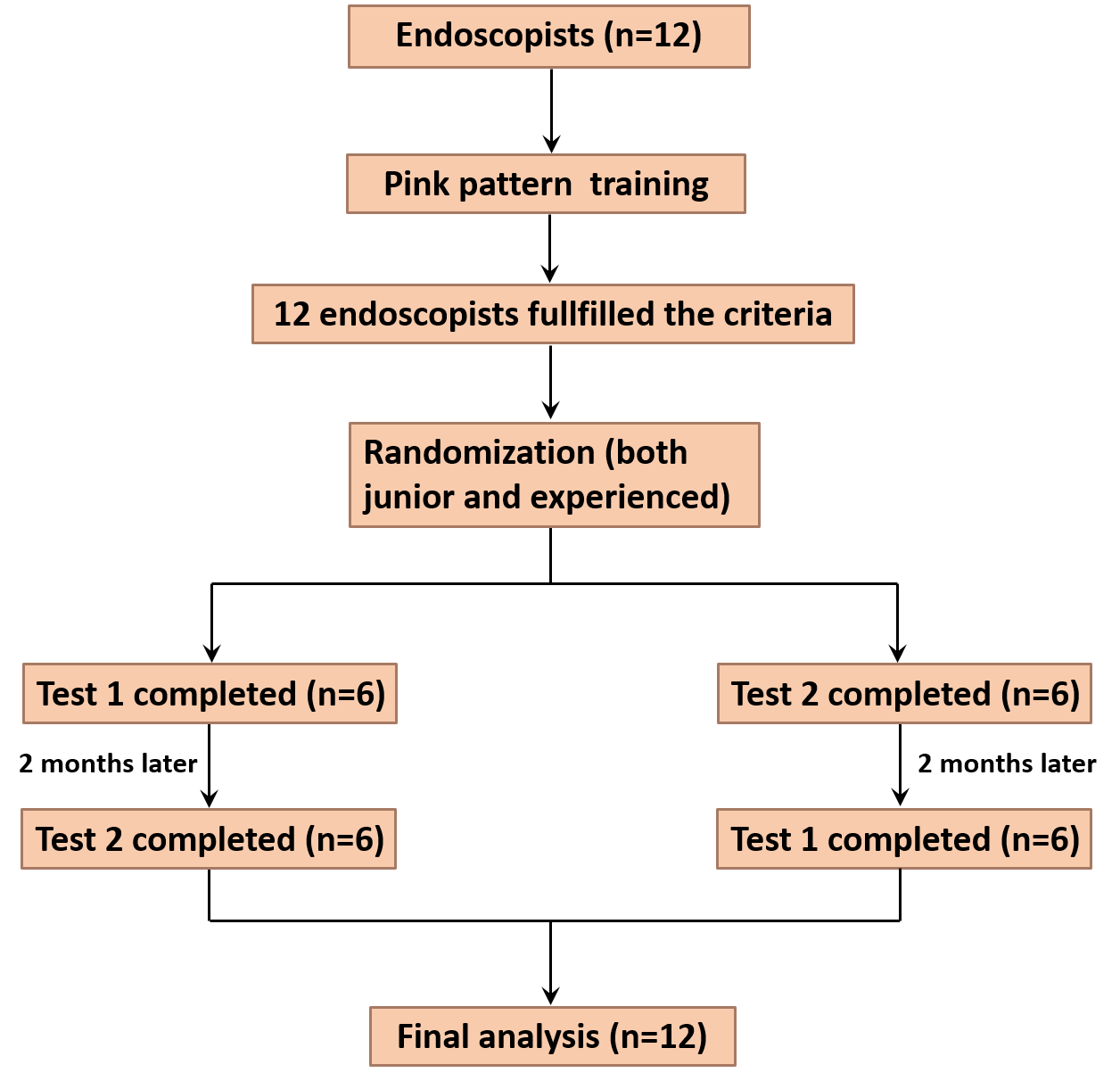


**Figure S4**


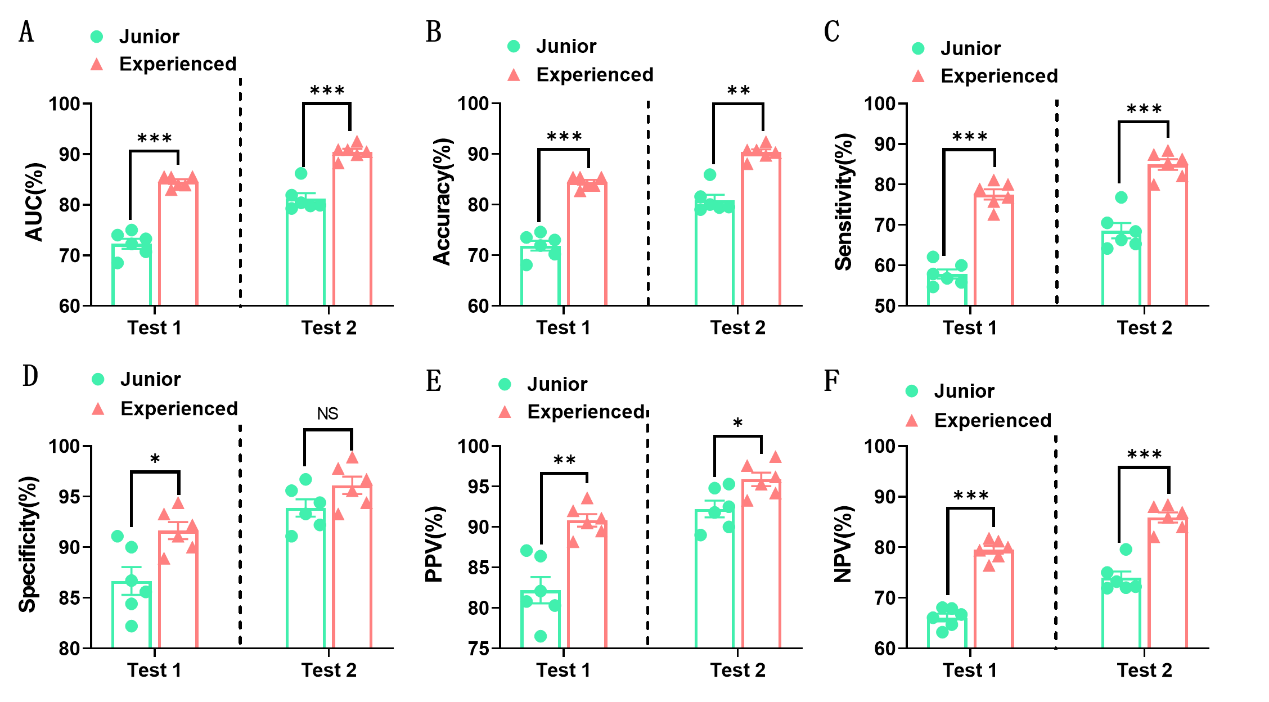


**Figure S5**
